# Monocyte unresponsiveness and impaired IL1β, TNFα and IL7 production are associated with a poor outcome in Malawian adults with pulmonary tuberculosis

**DOI:** 10.1186/s12879-015-1274-4

**Published:** 2015-11-13

**Authors:** Catriona John Waitt, Peter Banda, Sarah Glennie, Beate Kampmann, S. Bertel Squire, Munir Pirmohamed, Robert Simon Heyderman

**Affiliations:** Malawi-Liverpool-Wellcome Clinical Research Programme, University of Malawi College of Medicine, PO Box 30096, Chichiri, Blantyre Malawi; Department of Molecular and Clinical Pharmacology, The University of Liverpool, Block A, The Waterhouse Buildings, 1-5 Brownlow Street, Liverpool, L69 3GL United Kingdom; Department of Medicine, College of Medicine, University of Malawi, Blantyre, Malawi; School of Cellular and Molecular Medicine, University of Bristol, Bristol, UK; Imperial College London, London, UK; MRC Unit, The Gambia, Serrekunda, Gambia; Liverpool School of Tropical Medicine, Liverpool, United Kingdom

**Keywords:** Tuberculosis, Innate immunity, Cytokines, Monocytes, Africa, Clinical outcome

## Abstract

**Background:**

Early death during TB treatment is associated with depressed TNFα response to antigenic stimulation and propensity to superadded bacterial infection. Hypothesising the role of monocyte unresponsiveness, we further compared the immunological profile between patients who died or suffered a life-threatening deterioration ('poor outcome') during the intensive phase of TB treatment with patients who had an uneventful clinical course (‘good outcome’) who had been recruited as part of a larger prospective cohort study of Malawian TB patients.

**Methods:**

Using Luminex, IL1β, IL2, IL4, IL5, IL6, IL7, IL8, IL10, IL12, IL13, IL17, GCSF, GMCSF, MCP1, MIP1b, IFNγ and TNFα were measured in whole blood assay supernatants (stimulated with Mycobacterium tuberculosis H37Rv and LPS) and serum from 44 Malawian adult TB patients (22 of each outcome) immediately prior to commencing treatment, after 7 days and on day 56 of TB treatment. Monocyte surface expression of CD14, CD16, TLR2, TLR4, CD86 and HLADR, and intracellular TNFα were measured by flow cytometry as was intracellular TNFα response to purified TLR ligands.

**Results:**

Lower TB antigen-induced IL1β (*p* = 0.006), TNFα (*p* = 0.02) and IL7 (*p* = 0.009) were produced in the poor outcome group. TNFα was produced by ‘classical’ CD14^hi^CD16^lo^ monocytes, with no correlation between this response and expression of monocyte surface markers. Response to TB antigens correlated with responses to the purified TLR 2, 3 and 4 ligands.

**Conclusions:**

Dysregulated monocyte cytokine production was identified in TB patients with poor outcome. Lower TNFα responses to H37Rv paralleled lower responses to a panel of TLR ligands, suggesting an underlying perturbation in common TLR signalling pathways. Future work should explore the role of TLR polymorphisms in immune response and clinical outcome in TB patients.

**Electronic supplementary material:**

The online version of this article (doi:10.1186/s12879-015-1274-4) contains supplementary material, which is available to authorized users.

## Background

The global burden of tuberculosis (TB) remains considerable with an estimated 8.6 million new cases of TB and 1.3 million deaths in 2012. In a prospective cohort of 321 Malawian adults, we have recently shown that 12 % of laboratory confirmed pulmonary tuberculosis (PTB) cases either died or had an adverse event (principally related to TB or presumed secondary bacterial infection) during the first two months of treatment. In 50 % of HIV-negative and 63 % of HIV-positive TB patients, the cause of self-presentation with a life-threatening clinical event was a presumed superadded bacterial infection, with isolation of *Streptococcus pneumoniae* and *Salmonella typhiumurium* in a proportion. This poor outcome was associated with reduced production of the pro-inflammatory cytokine TNFα in response to stimulation with heat killed Mycobacterium tuberculosis H37Rv (H37Rv) and lipopolysaccharide (LPS) [1]. However, the molecular and cellular processes that underlie this association are unclear.

In acute bacterial sepsis, dysfunctional monocyte responses have been described where down-regulation of HLA-DR on CD14+ monocytes [2, 3] correlates with poor outcome [4] and a propensity for secondary bacterial infection [2, 4]. Decreased expression of the co-stimulatory molecule CD86 [5, 6] is also associated with greater severity of illness and inflammation in severe sepsis [7]. Monocyte recognition of pathogen associated molecular patterns (PAMPs) including bacterial and mycobacterial antigens occurs via conserved pathogen recognition receptors, among which the Toll Like Receptors (TLRs, principally TLR2, TLR4 and TLR9) have a major role. We therefore hypothesised that the depressed TNFα production and the susceptibility to secondary infection seen in our TB patients might represent an analogous process to that described in acute bacterial sepsis, and that inter-individual variability in TLR signalling might underpin this. We therefore sought to further characterize the cytokine and chemokine response profile in our cohort and investigate the relationship between monocyte immunophenotype, TLR utilization and clinical outcome.

## Methods

### Patient populations

This prospective cohort of Malawian pulmonary TB patients has been previously reported, comprising 199 patients with microbiologically proven disease (by sputum smear or culture). Median age was 31 (range 18–69), 61 % were male, 72 % were sputum smear positive and 60 % were HIV-positive with a median CD4 count of 150 (IQR 68–346) cells/mm^3^. [1]. Ethical approval for this study was granted by the College of Medicine Research Ethics Committee, University of Malawi (P.04/05/353) and by the ethics committee of the Liverpool School of Tropical Medicine (05.41). Written informed consent was obtained from all participants.

All patients were of Chewa descent, the most prevalent ethnic group in southern Malawi. Patients were categorised as ‘poor outcome’ if they died or suffered a life-threatening clinical deterioration necessitating urgent medical care during the two month intensive phase of TB treatment, and ‘good outcome’ if their clinical course was uncomplicated. The cytokine analysis was conducted on all 22 patients who suffered a poor outcome matched by age, sex, HIV status and CD4 count with 22 good outcome patients (summarized in Table 1).

**Table 1:** Key characteristics of cases and controls

Pair	Case/Control	Clinical Diagnosis	Sex	HIV	CD4 (cells/mm^3^)	BMI (Kg/m^2^)	Sputum Smear	Sputum Culture	Day of Event
1	Case (death)	Advanced TB and severe anaemia	F	Positive	44	17.8	Positive	Positive	14
1	Control		F	Positive	65	17.7	Positive	Positive	
2	Case (death)	Unknown (died at home)	F	Positive	92	13.4	Positive	Positive	31
2	Control		F	Positive	174	15.6	Negative	Positive	
3	Case (death)	Unknown (died at home)	F	Positive	333	20.4	Positive	Positive	7
3	Control		F	Positive	305	17.7	Negative	Positive	
4	Case (recovered)	Pneumonia	F	Positive	362	18.4	Negative	Positive	8
4	Control		F	Positive	344	19.6	Positive	Positive	
5	Case (death)	1 week postpartum and sudden collapse, possible pulmonary embolus	F	Negative	376	17.0	Positive	Positive	6
5	Control		F	Negative	400	19.5	Positive	Positive	
6	Case (death)	Septic shock, *Salmonella typhimurium*	M	Positive	16	14.2	Positive	Positive	3
6	Control		M	Positive	38	19.8	Positive	Positive	
7	Case (recovered)	Severe anaemia	M	Positive	19	18.4	Positive	Positive	11
7	Control		M	Positive	39	19.0	Positive	Positive	
8	Case (death)	*Salmonella typhimurium*	M	Positive	33	17.1	Positive	Unavailable	0
8	Control		M	Positive	72	14.1	Negative	Positive	
9	Case (death)	Unknown (died at home)	M	Positive	44	16.2	Positive	Positive	14
9	Control		M	Positive	81	16.0	Positive	Positive	
10	Case (death)	Pneumonia	M	Positive	50	14.7	Positive	Positive	20
10	Control		M	Positive	92	20.6	Positive	Positive	
11	Case (recovered)	*Salmonella typhimurium*	M	Positive	77	18.2	Positive	Positive	22
11	Control		M	Positive	93	19.0	Positive	Positive	
12	Case (recovered)	Gastroenteritis and hypovolaemic shock (Blood culture negative)	M	Positive	94	14.5	Positive	Positive	28
12	Control		M	Positive	112	21.6	Negative	Positive	
13	Case (death)	Septic-shock like presentation (Blood culture negative)	M	Positive	96		Positive	Positive	1
13	Control		M	Positive	150	15.3	Positive	Positive	
14	Case (death)	Pneumonia	M	Positive	105		Positive	Positive	40
14	Control		M	Positive	154	21.1	Positive	Positive	
15	Case (death)	Advanced TB	M	Positive	173	N/A	Positive	Unavailable	10
15	Control		M	Positive	183	20.1	Negative	Positive	
16	Case (recovered)	Severe anaemia	M	Positive	186	16.1	Positive	Positive	28
16	Control		M	Positive	208	20.9	Positive	Positive	
17	Case (death)	Advanced TB	M	Positive	200	19.6	Positive	Positive	53
17	Control		M	Positive	368	21.4	Negative	Positive	
18	Case (recovered)	Pneumonia	M	Negative	356	18.1	Positive	Positive	6
18	Control		M	Negative	325	18.9	Positive	Positive	
19	Case (recovered)	Pneumonia	M	Positive	399	19.9	Negative	Positive	6
19	Control		M	Positive	388	20.0	Positive	Positive	
20	Case (recovered)	Heptatotoxicity	M	Negative	403	14.9	Positive	Positive	15
20	Control		M	Negative	431	21.2	Positive	Positive	
21	Case (recovered)	Empyema – *Streptococcus pneumoniae*	M	Negative	426	14.8	Positive	Positive	38
21	Control		M	Negative	367	18.1	Positive	Positive	
22	Case (death)	Advanced TB	M	Negative	532	N/A	Positive	Unavailable	0
22	Control		M	Negative	484	19.8	Positive	Positive	

**Fig. 1:**
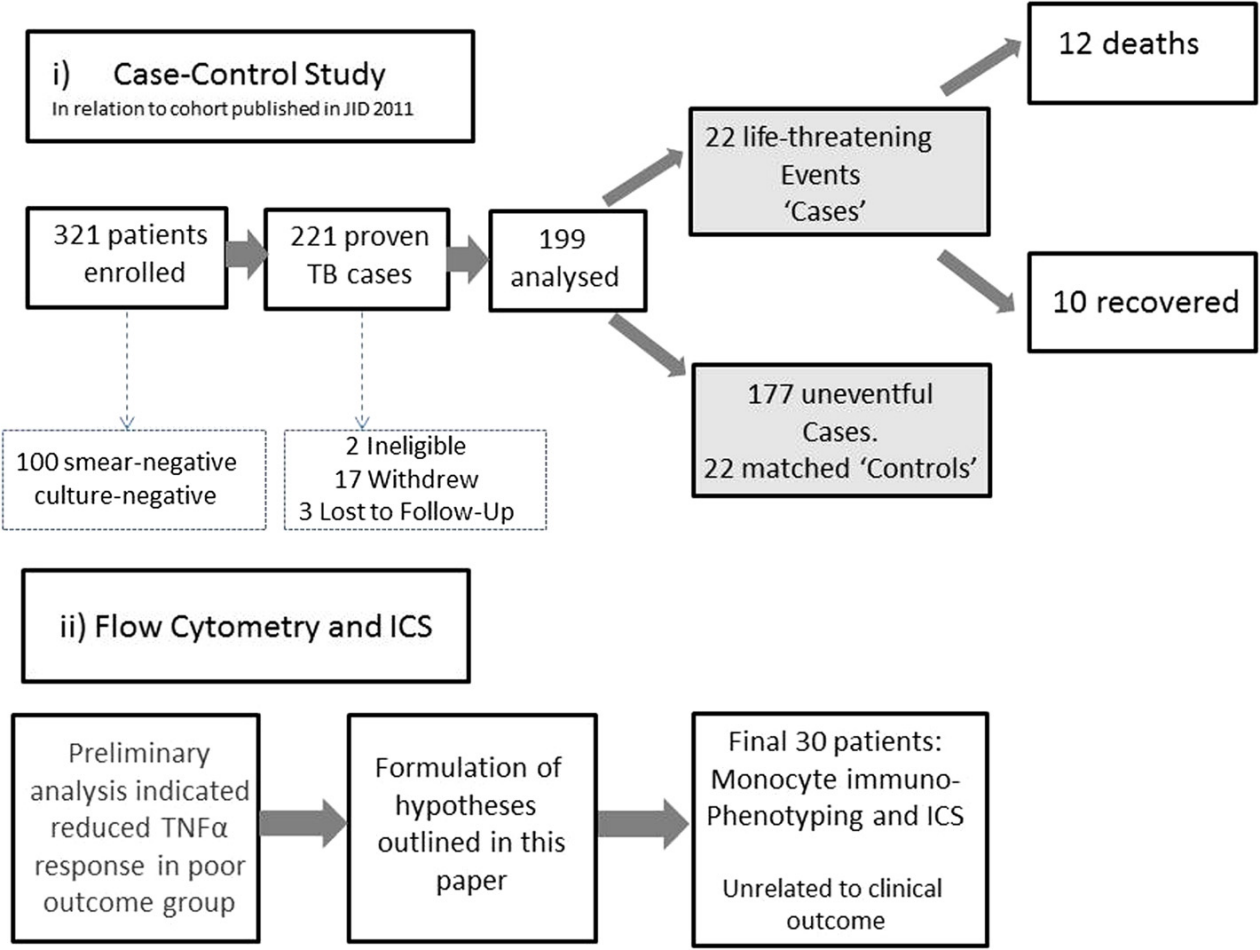
Flowchart indicating the relationship of i) the case–control study and ii) the flow cytometry and ICS study in relationship to the parent study (Waitt et al., JID 2011)

Following analysis of this cytokine data, monocyte immunophenotyping and intracellular cytokine staining assays were performed in real-time in a further cohort of 30 consecutive patients.. The selection of these populations in relation to the previously published work is summarised in Fig. 1.

### 17-plex analysis of antigen-induced and serum cytokines

The whole blood stimulation assay has previously been reported [1]. In brief, whole blood was dilulated (1:5, v/v) with serum-free media and stimulated for 16 h with either heat killed H37Rv or LPS. Supernatants were harvested and frozen at −80 °C prior to analysis. The antigen-induced cytokine concentration was calculated by subtracting the background concentration in the unstimulated control sample. The Bio-Plex Pro Human Cytokine 17-plex assay (IL1β, IL2, IL4, IL5, IL6, IL7, IL8, IL10, IL12, IL13, IL17, GCSF, GMCSF, MCP1, MIP1b, IFNγ and TNFα) was chosen to quantitate the majority of cytokines and chemokines with proven major roles in anti-mycobacterial immunity; other work has utilized the same panel [8]. This was performed according to the manufacturer’s recommended protocol (BioRad Laboratories, UK). Serum and stimulated whole blood assay [1] supernatants from day 0 and day 7 of TB treatment were analyzed to calculate the area under the curve (AUC) for cytokine production between these time points in attempt to quantify cytokine exposure during that time period. Additionally, to investigate cytokine dynamics throughout the intensive phase of TB treatment, serum and whole blood assay supernatants from six of the ten patients who suffered a life-threatening clinical deterioration and yet recovered to complete TB treatment were compared with matched controls on day 56 of TB treatment; matched data were unavailable for the remaining four patients who recovered clinically. Luminex data were acquired with a Bio-Plex System 200 (Bio-Rad, UK) and analyzed using Bio-Plex Manager 5.0 software (Bio-Rad, UK).

### Monocyte immunophenotyping

Whole blood was stained (FITC labelled anti-human CD16, PE labelled CD86 , PerCP labelled anti-HLA-DR and APC labelled anti-human CD14 [all BDSA]) and then incubated for 10 min prior to lysis (FACSLysing Solution, BD Biosciences), washing (PBS + 1%BSA, Sigma Aldrich) and fixation (2 % paraformaldehyde). Samples were kept refrigerated at 4 °C, protected from the light, prior to acquisition by flow cytometry (BD FACScalibur, BD Biosciences, Oxford, UK).

### Intracellular cytokine staining

Whole blood was incubated with H37Rv (American Type Culture Collection [ATCC No. 25618, Rockville, MD]), LPS (*E coli*, strain 055.b5 lipopolysaccharide, Sigma Aldrich) or TLR agonists (ultra pure E coli LPS [tlrl-pelps]- TLR4, Pam2CSK4 [tlrl-pam2] – TLR2, Pam3CSK4 [tlrl-pms] – TLR2/1 and Poly(I:C) [tlrl-pic] – TLR 3, Invivogen, Nottingham, UK) for 18 h with addition of 1 μl Brefeldin A (Sigma Aldrich) after the initial two hours. Following lysis, fixation and permeabilization steps (FACSLysing Solution, Cytofix/Cytoperm and Perm/Wash Buffer, all BD Biosciences), cells were stained with FITC labelled anti-human CD14, PE labelled anti-human IL10, PerCP labelled anti-human HLA-DR and APC labelled anti-human TNFα for 15 min. Data was acquired using a BD FACSCalibur (BD Biosciences, Oxford, UK). Monocytes were identified on the basis of their forward scatter-side scatter characteristics. A minimum of 100 000 PBMC events were analysed. The percentage positivity in the unstimulated control sample was subtracted from the stimulated samples.

### Statistical analysis

Luminex standards with a recovery outside the recommended boundaries of 70 – 130 % were excluded, as were those with a CV of greater than 15 %. The interpretation of values outside the detection range of the assay followed published methodology used in similar patient populations [9, 10], resulting in exclusion of antigen-induced production of IL5, IL6, IL8, MCP1, MIP1b and GMCSF from the analysis. Analysis therefore focused on the remaining 11 cytokines. Applying the Bonferroni correction for multiple comparisons gave a p-value for significance of 0.0045.

Flow cytometric data were analysed using FlowJo version 7.6.1 for Windows, under the licensing agreement for researchers in Africa provided by TreeStar Inc. Statistical analysis of data was performed using GraphPad Prism version 5.00 for Windows (GraphPad Software, San Diego California USA, www.graphpad.com).

Comparison of two matched results from an individual donor used the paired t-test. Analysis of non-parametric data used the Mann Whitney U test for comparison of medians. The direction and magnitude of correlation between responses to TLR ligands followed a normal distribution and therefore was analyzed by calculating the Pearson correlation co-efficient, whereas the Spearman correlation co-efficient described relationships between cytokine production and cell count which had a skewed distribution. In these analyses, statistical significance was present when *p* < 0.05.

## Results

### Cytokine and chemokine production in response to H37Rv and LPS

**Fig. 2:**
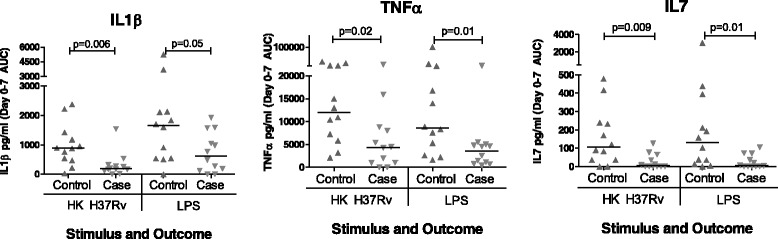
Cytokine responses to stimulation with H37Rv and LPS, expressed as the area under the concentration time curve (AUC) between Day 0 (immediately prior to commencing treatment) and Day 7 of TB treatment. Patients who had a poor outcome had lower production of TNFα, IL1β and IL7 compared to matched control patients who had an uneventful clinical course. No significant differences were identified between patient groups in the other cytokines and chemokines analysed

**Fig. 3:**
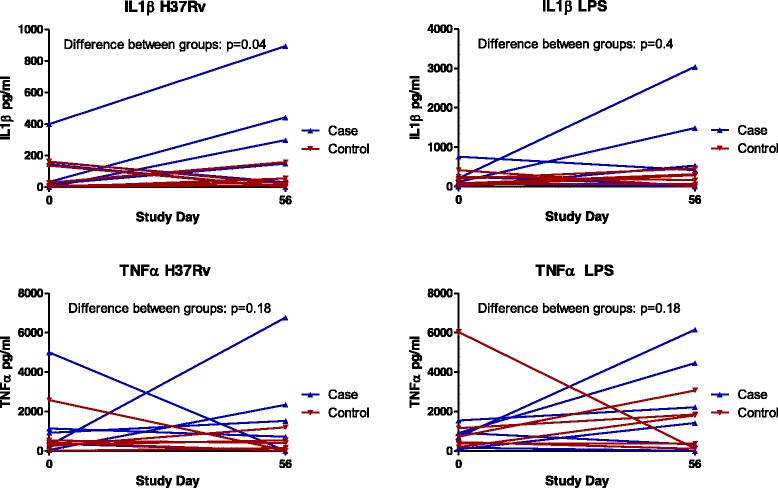
IL1β and TNFα production in response to stimulation with H37Rv and LPS immediately prior to the start of TB treatment and at the end of the intensive phase, in 6 patients who had a life-threatening clinical deterioration (poor) with controls matched by age, sex, HIV status and CD4 count who had an uneventful clinical course. There was significant restoration of the depressed IL1β responses in parallel with clinical recovery (*p* = 0.04), with a trend towards restoration of TNFα response (0.18)

Patients with poor clinical outcome had trends towards lower whole blood IL1β (median 196 [IQR 133–308] vs 894 [IQR 498–1368] *p* = 0.006 for H37Rv; median 630 [IQR 144–1447] vs 1661 [IQR 548–2134] *p* = 0.05 for LPS), TNFα (median 4355 [IQR 981–8598] vs 11977 [IQR 6221–22285] *p* = 0.02 for H37Rv; median 3512 [IQR 809–4927] vs 8623 [IQR 3309–19846] *p* = 0.01 for LPS) and IL7 (median 3.75 [IQR 0–57] vs 105 [IQR 37 – 479] *p* = 0.009 for H37Rv; median 4.64 [IQR 0–61] vs 131 [IQR 18.7 – 349] *p* = 0.01 for LPS) production in response to both H37Rv and LPS (Fig. 2) in comparison with patients who had an uneventful clinical course. No significant differences were identified in the levels of the other analytes (IL2, IL4, IL10, IL12, IL13, IL17, GCSF and IFNγ) measured in response to these agonists (see Additional file 1: Table S1 for a complete list of analyses performed), or in any of the panel measured in serum at the same time points. No statistically significant differences in cytokine profile were identified between the groups of patients when assessing the values at commencement of TB treatment alone. Follow-up of patients who survived a life-threatening deterioration until the end of the 56 day intensive phase of TB treatment revealed a trend towards restoration of the initially depressed IL1β (*p* = 0.04) and TNFα responses (*p* = 0.18) when compared with controls (Fig. 3).

i) Cellular source of TNFα production

**Fig. 4:**
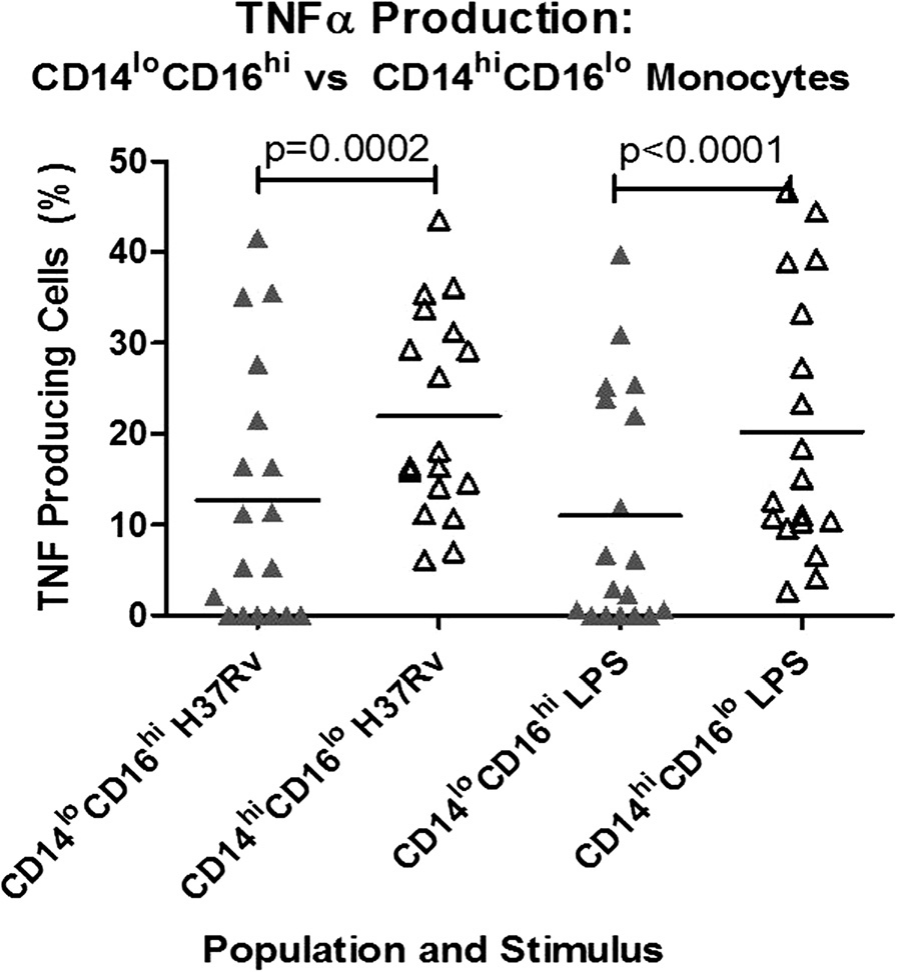
TNFα production in response to stimulation with H37Rv and LPS in ‘classical’ CD14^hi^CD16^lo^ and CD14^lo^CD16^hi^ monocyte populations. A significantly greater percentage of CD14^hi^CD16^lo^ cells produced TNFα in response to stimulation with H37Rv (*p* = 0.0002) and LPS (*p* < 0.0001)

Intracellular cytokine staining monocytes from TB patients revealed the predominant producer of TNFα in whole blood to be the ‘classical’ monocyte phenotype, staining strongly for CD14 and weakly for CD16 (CD14^hi^CD16^lo^ monocytes). Although we were able to identify the CD14^lo^CD16^hi^ cells which have been described in bacterial sepsis (Additional file 2: Figure S1) [11], the proportion of these cells producing TNFα in response to stimulation with H37Rv and LPS was much less (Fig. 4). There was no correlation between total TNFα production and absolute monocyte count (*r* = 0.11, *p* = 0.23 for the response to H37Rv; *r* = 0.03, *p* = 0.70 for the response to LPS).

ii) Monocyte Surface Markers

There was no correlation between monocyte expression of TLR2, TLR4, HLADR or CD86 with TNFα production (data not shown).

iii) Response to TLR Ligands

Although the expression of TLRs 2 and 4 on the cell surface was not associated with the magnitude of TNFα production, there were significant correlations between production of TNFα in response to H37Rv and LPS and the purified TLR ligands P3CSK (R^2^ = 0.78, *p* < 0.0001 for H37Rv; R^2^ = 0.69, *p* = 0.0001 for LPS), Poly(I:C) (R^2^ = 0.54, *p* = 0.0018 for H37Rv; R^2^ = 0.66, *p* = 0.0003 for LPS) and Ultrapure LPS (R^2^ = 0.52, *p* = 0.0024 for H37Rv; R^2^ = 0.59, *p* = 0.0008 for LPS) which act via these receptors and their downstream pathways (Table 2).

**Table 2:** Correlation of TNFα responses to TB antigens and TLR ligands

TLR agonist (18 h)		Corresponding TLR receptor (Spearman’s R and p-value)	
Ligand	Receptor	H37Rv (TB)	LPS (bacterial sepsis)
Ultrapure LPS	TLR4	0.52, *p* = 0.0024	0.59, *p* = 0.0008
Pam2CSK4	TLR2	0.16, not significant	0.38, *p* = 0.01
Pam3CSK4	TLR1/2	0.78, *p* < 0.0001	0.69, *p* = 0.0001
Poly(I:C)	TLR3	0.54, *p* = 0.0018	0.66, *p* = 0.0003

## Discussion

Having previously shown that early deaths during tuberculosis treatment are associated with depressed innate immune responses, bacterial infection and tuberculosis progression, we have now demonstrated this to be a consequence of broad-ranging monocyte hyporesponsiveness to pathogenic stimulation. In addition to the lower TNFα in response to both LPS and heat-killed Mycobacterium Tuberculosis H37Rv already described [1], we noted lower IL1β and IL7 production in the poor outcome group. IL1β is a pro-inflammatory cytokine involved in the initial response to tuberculosis [12]. Production is suppressed by IL4 [13] and IL10 [14]; however we did not see changes in these regulatory cytokines in our cohort. IL7 is a haemopoietic growth factor secreted predominantly by stromal cells in the bone marrow and thymus, and to a lesser degree keratinocytes, dendritic cells, hepatocytes, neurones and epithelial cells. It has a key role in T cell homeostasis with perturbation of the IL7/IL7 receptor axis occurring with advancing HIV but the biological role of IL7 in pulmonary tuberculosis is uncertain. Lower levels have been reported in advanced disease [15] and reduced expression of IL7Rα was found in patients with active versus latent disease [16]. Patients who recovered from a life-threatening event revealed a trend towards recovery in IL1β and TNFα responses, suggesting the initial dysfunction to be a transient phenomenon. Analogous to this may be the finding that endotoxin-tolerant monocytes from patients with severe sepsis reverted to normal responsiveness following successful treatment, in contrast to cells from patients who died, which never recovered [17].

We therefore sought to determine whether the monocyte dysfunction in TB mirrored that reported in sepsis. However, despite some similarities, several important differences were seen. Firstly, whereas an expanded subset of CD16 monocytes has been described as a major producer of TNFα in response to stimulation with both LPS and the TLR ligand P3CSK [11] in patients with bacterial sepsis, the expanded population of CD14^lo^CD16^hi^ cells in TB patients had little functional relevance in terms of TNFα production. Secondly, in contrast to the decreased monocyte HLA-DR expression which has been proposed as a potential prognostic marker in patients with sepsis [18, 19], we found variable HLA-DR expression which did not correlate with functional capacity to produce TNFα or clinical outcome. Whilst expression of CD86 positively correlated with HLA-DR, we identified no functional significance of this molecule in relation to TNFα production.

Not having explained variability in TNFα production in terms of monocyte surface markers with proven significance in bacterial sepsis, we next investigated the contribution of TLR signalling to TNFα response. Among the TLRs, TLR2, TLR4 and TLR9 have proven roles in the recognition of, and response to, *M tuberculosis*. Purified mycobacterial antigens preferentially interact with TLR2, in combination with TLR1 and TLR6. However, infection with whole bacilli evokes a more complex interaction pattern involving at least TLR2 and TLR4 and leads to differential activation of antibacterial effector pathways, which is MyD88 signalling dependent [20]; studies investigating the role of individual TLRs in response to mycobacterial antigens are not fully conclusive [21]. TLR9, which is located intracellularly, responds to antigens following phagocytosis [21]. Ligation of TLRs triggers a cascade of intracellular events, initiated by the activation of the adapter molecule MyD88. Finally, translocation of the transcription factor NFκB to the nucleus provides the signal for up regulation of cytokines, chemokines and co-stimulatory molecules which shape the subsequent development of immune responses. Whilst monocyte expression of TLRs 2 and 4 during the intensive phase of TB treatment did not correlate with clinical severity or overall TNFα production, use of purified TLR ligands demonstrated reduced functional capacity of these receptors in terms of triggering the expected inflammatory response to pathogenic stimulation. The depressed TNFα production in response to HKH37Rv and LPS paralleled decreased responses to TLR1, 2, 3, 4 and 6 ligands, suggesting that rather than a pathogen-specific process, a generalised innate hyporesponsiveness is present. Although many investigators have focussed on the relationship between specific mycobacterial antigens and individual TLRs, it has recently been suggested that the downstream processes involving the adaptor molecule MyD88 may be of greater clinical relevance [21, 22]. Our findings are consistent with this hypothesis, suggesting the occurrence of a generalised dysfunction of TLR signalling rather than a defect in a specific receptor pathway. Conversely, a recent genome-wide association study conducted in a healthy Caucasian population identified the TLR10/1/6 locus as dominant common genetic factor influencing inter-individual variability in TLR-induced whole blood responses [23]; the significance of this in human disease states warrants further evaluation. Table 3 summarises the key differences between our findings in TB patients and those which have been reported in patients with sepsis.

**Table 3:** Comparison of immune dysregulation between bacterial sepsis and tuberculosis

Bacterial Sepsis	Malawian TB Patients
↓monocyte HLA-DR expression associated with secondary infection [2, 4]	Variable monocyte HLA-DR expression^a^
HLA-DR expression correlates with TNFα production [24]	No correlation between monocyte HLA-DR expression and TNFα production
Correlation between monocyte HLA-DR and CD86 expression [6, 25]	Correlation between monocyte HLA-DR and CD86 expression
↓CD86 expression associated with increased severity of inflammation [7, 25]	Widely variable monocyte CD86 expression^a^
Expansion of CD14^lo^CD16^hi^ monocyte population [26]	Expansion of CD14^lo^CD16^hi^ monocyte population
CD14^lo^CD16^hi^ monocytes major TNFα producers [11]	‘Classical’ CD14^hi^CD16^neg^ monocytes major TNFα producers

^a^Sample size insufficient to correlate with clinical outcome

### Limitations

This study was limited by the identification of only 22 patients who died or had a life-threatening event during the intensive phase of TB treatment. However, this study was able to investigate both serum and antigen-induced cytokine responses in association with clinical outcome in a longitudinal cohort of TB patients containing both HIV positive and negative individuals, in contrast with previous studies which have tended to be cross sectional and more specific in their inclusion criteria. Additionally, the small number of patients included in the flow cytometric analysis precluded correlation with clinical outcome; this was unavoidable as these analyses could not be performed on stored samples. Nonetheless, the results presented here will inform the design of larger prospective immunological studies.

## Conclusions

Malawian adults who clinically deteriorated during the intensive phase of TB treatment had trends towards lower monocyte derived cytokine responses (IL1β, TNFα and IL7) to antigenic stimulation than matched patients with good clinical outcome. Although validation of these findings in an unrelated cohort of TB patients from another setting is necessary, our data suggest this relates to a generalised down-regulation of TLR signalling pathways rather than a defect in a specific receptor. Further work investigating these pathways may help explain the vulnerability to superadded infection which is a major cause of poor clinical outcome in this population.

## Additional files

Additional file 1: Table S1. Median and IQR for responses to stimulation with heat killed Mycobacterium Tuberculosis (H37Rv) or Lipopolysaccaride (LPS). P-values are derived from Mann-Whitney U tests. 11 analyses were performed for each stimulus, giving a corrected p value of 0.0045 using the Bonferroni correction. (DOCX 17 kb) Additional file 2: Figure S1. Gating strategy to identify monocyte populations. The first plot represents the forward scatter- side scatter characteristics and the second the separation according to CD14 and CD16 staining. (JPEG 61 kb)
