# Supplementary material for: Monocyte unresponsiveness and impaired IL1β, TNFα and IL7 production are associated with a poor outcome in Malawian adults with pulmonary tuberculosis
Source: BMC Infect Dis. 2015 Nov 13;15:513. doi: 10.1186/s12879-015-1274-4 (PMC4643523; doi:10.1186/s12879-015-1274-4)
Supplement: Additional file 1: Table S1. — Median and IQR for responses to stimulation with heat killed Mycobacterium Tuberculosis (H37Rv) or Lipopolysaccaride (LPS). P-values are derived from Mann-Whitney U tests. 11 analyses were performed for each stimulus, giving a corrected p value of 0.0045 using the Bonferroni correction. (DOCX 17 kb) [file 12879_2015_1274_MOESM1_ESM.docx]

Supplementary Table 1

|  | Response to heat-killed H37Rv | | | Response to LPS | | |
| --- | --- | --- | --- | --- | --- | --- |
| Cytokine | Good Outcome | Poor Outcome | p-value | Good Outcome | Poor Outcome | p-value |
| IL1β | 894 (498-1368) | 196 (133-308) | 0.006 | 1661 (548-2134) | 630 (143-1447) | 0.049 |
| IL2 | 49 (32-122) | 83 (0-130) | 0.93 | 8.2 (0-55) | 7.1 (0-105) | 0.64 |
| IL4 | 30 (22-51) | 25 (6-33) | 0.42 | 25 (18-43) | 15 (5-37) | 0.48 |
| IL7 | 105 (37-237) | 3.7 (0-56) | 0.009 | 131 (19-349) | 4.6 (0 – 61) | 0.010 |
| IL10 | 366 (151-655) | 385 (209-1000) | 0.58 | 1237 (394-2313) | 674 (240-1809) | 0.44 |
| IL12 | 0 (0-20) | 32 (0-86) | 0.073 | 56 (6.8-119) | 128 (27-284) | 0.24 |
| IL13 | 4.4 (0-258) | 0 (0-55) | 0.51 | 26 (0-242) | 0 (0-34) | 0.18 |
| IL17 | 395 (157-609) | 326 (195-733) | 0.88 | 219 (82-871) | 333 (7.3-1124) | 0.91 |
| IFNγ | 2852 (1775-6899) | 2571 (917-5623) | 0.40 | 2583 (1183-4726) | 1473 (353-4235) | 0.26 |
| TNFα | 11977 (2121-22285) | 4355 (981-8598) | 0.023 | 8623 (3309-19846) | 3512 (809-4927) | 0.012 |
| GCSF | 2157 (1004-6334) | 1372 (983-4550) | 0.58 | 1513 (803-4680) | 2070 (621-2882) | 0.71 |

Median and IQR for responses to stimulation with heat killed Mycobacterium Tuberculosis (H37Rv) or Lipopolysaccaride (LPS). P-values are derived from Mann-Whitney U tests. 11 analyses were performed for each stimulus, giving a corrected p value of 0.0045 using the Bonferroni correction.
