# Supplementary figures and images for: Monocyte unresponsiveness and impaired IL1β, TNFα and IL7 production are associated with a poor outcome in Malawian adults with pulmonary tuberculosis
Source: BMC Infect Dis. 2015 Nov 13;15:513. doi: 10.1186/s12879-015-1274-4 (PMC4643523; doi:10.1186/s12879-015-1274-4)

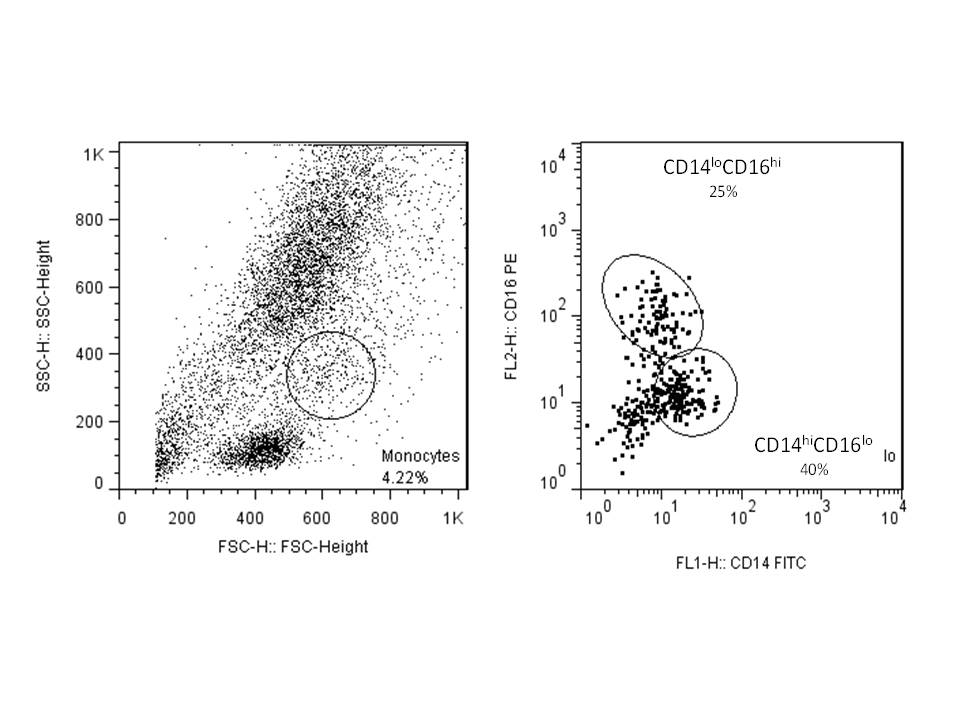

Supplement: Additional file 2: Figure S1. — Gating strategy to identify monocyte populations. The first plot represents the forward scatter- side scatter characteristics and the second the separation according to CD14 and CD16 staining. (JPEG 61 kb) [file 12879_2015_1274_MOESM2_ESM.jpeg]
